# Effects of Milk Storage Temperature at the Farm on the Characteristics of Parmigiano Reggiano Cheese: Chemical Composition and Proteolysis

**DOI:** 10.3390/ani11030879

**Published:** 2021-03-19

**Authors:** Piero Franceschi, Massimo Malacarne, Paolo Formaggioni, Michele Faccia, Andrea Summer

**Affiliations:** 1 Department of Veterinary Science, University of Parma, Via del Taglio 10, I-43126 Parma, Italy; piero.franceschi@unipr.it (P.F.); andrea.summer@unipr.it (A.S.); 2 Department of Soil, Plant and Food Sciences, University of Bari, Via Amendola 165/A, I-70125 Bari, Italy; michele.faccia@uniba.it

**Keywords:** Parmigiano Reggiano cheese, milk storage, temperature of storage, chemical composition, cheese ripening, nitrogen fractions

## Abstract

**Simple Summary:**

Parmigiano Reggiano is a Protected Designation of Origin (PDO) hard-cooked cheese made from raw milk that is ripened from 18 to over 24 months. Since the EU official protocol does not allow farmers to store the milk at the farm at a temperature lower than 18 °C, the conditions applied during storage strongly affect milk quality and suitability to cheese-making. At this temperature, the bacterial growth and the activity of proteolytic enzymes, responsible for milk alteration, cannot be inhibited. The aim of this research was to study the effect of storing milk at 9 °C, at the farm, on the chemical characteristics, proteolysis and organic acid profile of Parmigiano Reggiano cheese during ripening. The obtained results revealed lower fat and higher protein contents with respect to the cheese produced with milk stored at 20 °C. Regarding proteolysis, peptone nitrogen was the only parameter that significantly increased in the cheese produced with the milk stored at 9 °C. Despite of the statistical significance of the differences observed, they appeared as too small for changing the overall “typicality” of the cheese, and should be included within the ordinary range of variability of Parmigiano Reggiano.

**Abstract:**

Parmigiano Reggiano is a Protected Designation of Origin (PDO) cheese whose official production protocol provides that milk cannot be stored at less than 18 °C at the farm. The possibility of refrigerating milk at the farm is highly debated, since it should allow for the limiting of bacterial growth, thus improving the quality of the cheese. The present research aimed to study the influence of storing the milk at 9 °C on the chemical composition and proteolysis during the ripening of Parmigiano Reggiano cheese. The experimentation considered six cheese-making trials, in which both evening and morning milks were subdivided into two parts that were maintained at 9 and 20 °C. After Parmigiano Reggiano cheese-making, one of the twin wheels obtained was analyzed after 21 months of ripening. From each cheese, two different samples were taken, one from the inner zone, and the other from the outer zone. The results of the chemical analyses evidenced that milk storage at 9 °C significantly (*p* ≤ 0.05) influenced fat, crude protein, soluble nitrogen and peptone nitrogen contents. Nevertheless, the differences observed with respect to the cheese obtained with milk stored under standard condition were very small and should be considered within the “normal variations” of Parmigiano Reggiano chemical characteristics.

## 1. Introduction

Parmigiano Reggiano is a Protected Designation of Origin (PDO) hard-cooked cheese with a long ripening period, ranging from a minimum of 18 to over 24 months. It is made from raw milk, by following a strict manufacturing protocol [1], and, for this reason, the microbiological characteristics of milk play a fundamental role on the yield and quality of the finished product [2,3]. According to the protocol, the cows must be milked two times a day, and the milk for cheese-making must be obtained by pooling the evening milk, partially skimmed by the natural creaming, with the whole morning milk [4].

The two most important hygienic parameters of milk are the somatic cells and total bacterial counts, whose increase beyond certain limits also causes worsening of the technological properties [5,6,7,8]. The increase of somatic cell count is associated with increase of proteolytic activity [9,10], which is responsible for the early degradation of casein in milk, loss of fat and protein during cheese-making [11] and consequent worsening of the cheese yield [9,12]. High values of bacterial count are associated with increased risk of the presence of bacterial species unsuitable for cheese-making, which cause defects in the cheese [13,14], with consequent depreciation of the product [2,5], and threaten its safety for human consumption.

Effective cooling of the milk at the farm is the only way to limit the bacterial growth and to slow down the activity of proteolytic enzymes, thus reducing the risk of milk alteration. On the other hand, refrigeration negatively influences the technological features of the milk, as it causes the dissociation of β-casein and inorganic calcium from the casein micelle, with negative repercussions on the rennet coagulation properties and cheese yield [13,15,16]. Furthermore, the low temperature favors the growth of psychotropic bacteria, whose strong proteolytic and lipolytic activities [14,17,18] are responsible for the arising of flavor defects during cheese ripening [19,20,21].

For this reasons, Parmigiano Reggiano regulation strictly disciplines the management of milk at the farm and requires that the time between the beginning of milking and delivery to the cheese factory must be shorter than 7 h. During this time (that is called maturation phase), the milk can be cooled, but temperature must be maintained above 18 °C [1].

The effects of such maturation phase on the chemical and microbiological characteristics of milk [22,23] and the consequences on the characteristics of vat milk for Parmigiano Reggiano manufacturing need further study [24]. Any decrease of coagulable casein [24], with connected reduction of yield [3] and worsening of the microbiological characteristics [13], should also influence the cheese-ripening process, with negative repercussions on cheese quality.

The most important biochemical event that occurs during cheese ripening is proteolysis. A common parameter used for its quantification is the ripening index, which represents the amount of casein that is hydrolyzed during the ripening process [25,26]. The main proteolytic enzymes involved in the proteolysis process of Parmigiano Reggiano cheese arise from milk (endogenous proteases and from adventitious micro-organisms), rennet, thermophilic lactic bacteria added with the natural whey starter, and somatic cells [27,28]. Their activity is influenced by the processing conditions that are applied during cheese-making and ripening. 

As the Parmigiano Reggiano cheese wheel is very large (about 23 cm high, about 43 cm in diameter and about 40 kg weight) and is molded at about 50 °C after the strong curd cooking phase, a temperature gradient is present in the different zones of cheese that last about 36 h [29]. Moreover, after brining, salt tends to concentrate in the outer zone and takes about 12 months to diffuse in the inner parts and reach the equilibrium. These phenomena determine different environmental conditions (in terms of temperature, salt concentration and water activity) in the different zones [26], which can both influence the enzymatic and microbial activities during ripening [19,29,30]. 

The aim of this research was to study the effect of keeping milk at the farm at 9 °C versus 20 °C on the chemical characteristics, proteolysis and organic acid profile of Parmigiano Reggiano cheese during ripening. Since the storage conditions at the farm were expected to exert a different effect on enzymatic and microbial profile in the cheese, the investigation was performed in different parts of the cheese wheel.

## 2. Materials and Methods 

### 2.1. Experimental Design, Sampling Procedure and Classification of Cheese Batches

**Figure 1:**
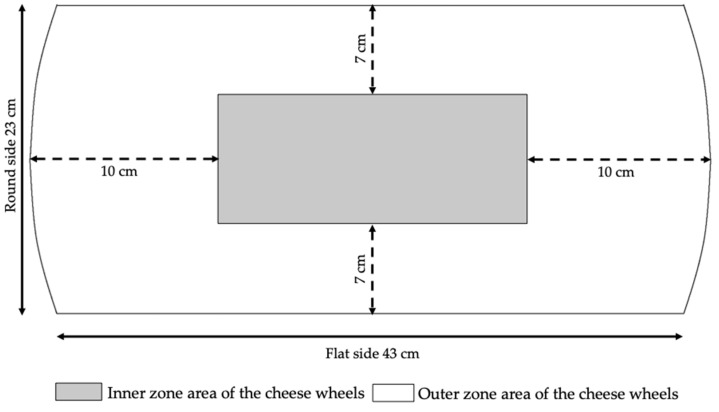
Scheme of sampling from a vertical section of a Parmigiano Reggiano cheese wheel.

Six cheese-making trials were performed, 3 in winter and 3 in summer. For each of them, both evening and morning milk deriving from a herd raising Italian Friesian cows were split, immediately after milking, into two different refrigerating tanks. In the first tank, milk was cooled and kept at 9 °C until delivery (MC9-milk); in the second one, it was cooled and kept at 20 °C (MC20-milk). In full compliance with the official production protocol [1], all milks were milked within about 3 h and delivered to the cheese factory within 1 h. Upon arrival, they were immediately submitted to cheese-making. For each trial, one of the two cheese wheels produced from the same vat was destined for the experimentation and sampled at 21 months of ripening (middle ripening time). Sampling consisted in taking two representative subsamples of the inner and outer parts, according to the method proposed by Malacarne et al. [31]: In short, the wheel was vertically cut from the center to the heel, in order to obtain a 4 cm thick section (Figure 1); then the rind was removed (5–7 mm), and two sub-samples, representative of the two zones, were obtained. Each sample was grated, stored at 4 °C and analyzed within 2 days.

### 2.2. Cheese-Making Process

Cheese-making was performed according to the official protocol of Parmigiano Reggiano PDO cheese [1], except for the cooling temperature of MC9-milk.

The full cream evening milk, immediately after arriving at the cheese factory, was placed into the creaming tank, where fat globules surfaced naturally during the night (it required approximately 12 h). Such “natural creaming” took place at room temperature. The following morning, the partially skimmed milk was extracted from the bottom of the creaming tank and transferred into the cheese-making vat. Here, the full cream morning milk arrived from the farm was added: this commingling constituted the so-called “vat milk” (containing about 2.6% fat and about 1–1.1 fat-to-casein ratio). Successively, a natural whey starter culture was added (2.5–3 L 100 kg ^−1^ milk), obtained by spontaneous acidification at room temperature of residual whey deriving from the cheese-making of the previous day. The inoculated vat milk was heated to 33 °C and clotted by adding calf rennet (1:120,000 strength; 2.5–3 g 100 kg^−1^ milk). After coagulation (10–12 min), the curd was cut into small grains and cooked at 55 °C. Then the curd particles were left to settle naturally on the bottom of the vat, to form a compact cheese mass. During this time (approximately 1 h), the temperature was kept constant at about 55 °C. The cheese mass was then extracted from the vat and divided into two parts which were placed into the molds, to form two twins cheese wheels. The cheeses were left to cool at room temperature, in the molds, for two days. During this period, the molds were periodically turned over, in order to promote a homogeneous distribution of the whey into the wheels. The cheeses were then placed in a saturated brine, for a period of 18–20 days, and finally they entered into the ripening room, where they remained for 21 months, in controlled environmental conditions (temperature of the room was between 16 and 18 °C, and value of relative humidity was 80%).

### 2.3. Analytical Methods

Total nitrogen (TN) content was determined by the Kjeldahl method [32], as well as the contents of soluble nitrogen at pH 4.4 (SN) [33], soluble nitrogen in 12% (*w*/*v*) trichloroacetic acid (NTCA) and soluble nitrogen in 10% (*w*/*v*) phosphotungstic acid (NPTA, corresponding to free amino acids nitrogen). Reagents for obtaining the nitrogen fractions were from Carlo Erba, Milan, Italy). Kjeldahl analysis was performed, using a DK6 Digestor and an UDK126A Distiller (VELP Scientifica, Usmate, Italy). In addition to the nitrogen fractions, the ammonia nitrogen (NNH_3_) was also determined by the method proposed by Savini [34]: in short, 10 g of grated cheese was homogenized by Ultraturrax (model T25, IKA, Merk, Darmstadt, Germany) in 50 mL of water, at 40 °C, and transferred quantitatively, using some more water at 40 °C, for rinsing, in a Kjeldahl test tube with 5 g of magnesium oxide (MgO, Carlo Erba Reagents, Milan, Italy), and then submitted to distillation within 30 min, in a steam distillation unit UDK126A Distiller (VELP Scientifica, Usmate, Italy). The distilled solution was collected in 20 mL sulphuric acid 0.1 N and titrated with sodium hydroxide 0.1 N (Carlo Erba Reagents, Milan, Italy). From the whole nitrogen-fractionation process, the values of crude protein (TNx6.38/1000), nitrogen of peptones (NS-NTCA), nitrogen of peptides (NTCA-NPTA-NNH_3_) and ripening index (NSx100/TN) were calculated [33].

Cheese fat content was determined by the volumetric Gerber method [35]; pH was measured directly in the cheese, with a specific potentiometer (Crison Instruments, Barcelona, Spain); sodium chloride (NaCl) was determined by titration with AgNO_3_ [36]. Dry matter (DM) was measured by oven-drying at 102 °C [37], and from this value, moisture was calculated (100-DM). Ash was determined by muffle calcination at 530 °C of a 2.5 g sample [38], and from ash dissolved in hydrochloric acid (2N; Carlo Erba Reagents, I-20010 Milan, Italy), as described by Malacarne et al. [39]; potassium, calcium and magnesium were determined by Atomic Absorption Spectrometry (Perkin-Elmer 1100 B, Waltham, MA 2451, USA), whereas phosphorus was determined by colorimetric method [40]. In short, 10 mL of hydrochloric ash solution diluted 40 time was placed in a test tube, with 2 mL of perchloric acid, 2 mL of a 2% (*w*/*v*) solution of 2:4 diamino-phenol-hydrochloride and 20% (*w*/*v*) of sodium metabisulfite and 1 mL of 8.3% (*w*/*v*) solution of ammonium molybdate (Carlo Erba Reagents, I-20010 Milan, Italy). After 25 min, 1 mL of this solution was read at 750 nm, with a spectrophotometer Helios γ (Thermo Fisher Scientific, Waltham, MA, USA).

The organic acids profile (acetic, propionic, lactic, pyruvic, citric, malic, fumaric and pyro-glutamic) was determined by chromatography as reported by Careri et al. [41].

D-lactic acid content was determined by using an enzymatic method based on reduction of D-lactic acid to pyruvate acid by D-lactate dehydrogenase enzyme [42] by means of a specific kit [43] (Steroglass, Perugia, Italy). L-lactic acid content was calculated by difference from total lactic acid content.

For fat, protein and sodium chloride, the value was expressed on dry matter, as follows:

CDM = CC × 100/DM,(1)

where CDM is the content on a dry matter basis, expressed as g/100 g of dry matter; CC is the content in the cheese, expressed as g/100 g cheese; and DM is the value of cheese dry matter.

Moreover, the ratios between the nitrogen (N) of peptones, peptides amino acids and ammonia and TN or SN in cheese were calculated as follows:

PV = NX × 100/NT,(2)

PV = NX × 100/NS,(3)

where PV is the percentage value of N of peptone, peptide, amino acids and ammonia; NX is the content (g/100 g) of peptones, peptides, amino acids and ammonia N; TN and SN are the contents of total and soluble nitrogen (g/100 g cheese), respectively.

Lactose, fat, protein and casein milk contents were determined by mid-infrared method [44], using a MilkoScan FT 6000, (Foss Electric, Hillerød, Denmark). Titratable acidity and pH values of milk were measured by potentiometer and titration of 50 mL of milk with 0.25 N sodium hydroxide with Soxhlet–Henkel method [45], respectively, both by Crison Compact Titrator D (Crison Instruments, Barcelona, Spain). Somatic cells and total bacterial count of milk were determined by using the fluoro-opto-electronic method [46] with Fossomatic (Foss Electric, Hillerød Denmark) and using the flow cytometry method [47] with BactoScan FC (Foss Electric, Hillerød, Denmark), respectively.

For microbiological analyses, serial decimal dilutions of milk were prepared and plated on the following media and incubation conditions:

– MRS agar (Oxoid) at 21 °C, for 5 days, for the count of mesophilic lactic acid bacteria [48].
– PCA (Oxoid) at 6.5 °C, for 10 days, for enumeration of psychrotrophic bacteria [49].
– Litmus milk (BD Diagnostics, Sparks, MD, USA) at 30 °C for 48 h for the enumeration of proteolytic bacteria.
– Tributyrin agar (Oxoid) at 30 °C, for 7 days, for lipolytic bacteria [50].

MRS agar (Oxoid) was also used for counting mesophilic lactic acid bacteria in cheese, by plating a diluted suspension of 10 g of grated cheese in 90 mL of 20% of trisodium citrate (pH 7.5) [48,51].

Finally the number of Clostridia spores was determined on milk by the Most Probable Number (MPN) method, after incubation for 7 days [52].

### 2.4. Statistical Analysis

The significance of the differences between the cheeses produced with milk kept at the farm at 9 and 20 °C was tested by analysis of variance and the least square means were calculated, using a general linear model procedure of software SPSS version 25 (IBM SPSS Statistics version 25, Armonk, NY, USA). The outer and inner part of the cheese were analyzed separately.

To this aim, various factors have been tested, including the effect of the season of sampling (two levels; winter and summer), the effect of the cheese factory (two levels, i.e., one level for each cheese factory) and the effect of the herd (two levels, i.e., one level for each cheese factory). However, when the effect of the trial is included in the model, all the other factors tested result not statistically significant.

Than the least square means are consequently calculated according to the following univariate model:

Y_ijk_ = µ + C_i_ + T_j_ + ε_ijk_, (4)

where Y_ijk_ = dependent variable; µ = overall mean; C_i_ = effect of the temperature, 9 or 20 °C (i = 1, 2); T_j_ = effect of trial (j = 1, ..., 6); and ε_ijk_ = residual error. The significance of the differences was tested by the Bonferroni method.

## 3. Results and Discussion

### 3.1. Effect on Milk and Cheese Chemical Composition

The chemical composition and microbiological profile of the full cream evening milk, stored at the herd at 9 or 20 °C, are shown in Table 1. Microbiological analysis was carried out only on the evening full cream milk before the natural creaming, as this process causes a strong reduction of bacterial and cell counts, as reported by Malacarne et al. [4].

Both chemical and microbiological parameters were not statistically different between the two experimental theses, except for psychrotrophic bacteria, whose count resulted higher (*p* ≤ 0.05) in MC20-milk than in MC9. Such a difference was probably due to the fact that the optimum of growth of psychrotrophs is just around 20 °C or even more [53,54]. Anyway, the count values were below the level of 5.11 log FCU/mL that Yuan et al. [55] reported to be detrimental for cheese-making. Over this concentration, indeed, these microorganisms are capable of producing hydrolytic enzymes that cause problems, such as the decrease of the cheese-making yield and formation of off-flavors in the ripened cheese; in the case of raw-milk cheeses, these enzymes accumulate until the curd cooking [54].

The values of the chemical and microbiological parameters were in agreement with those reported by Coloretti et al. [56]. On the same type of milk, these authors found 3.58 g/100 mL fat, 2.55 g/100 mL casein, 5.54 log10 somatic cells/m, 4.85 log10 CFU/mL total mesophilic bacteria and 3.30 log_10_ CFU/mL mesophilic lactic acid bacteria. Microbiological data at 9 °C were compared with those recorded by Malacarne et al. [13] under similar storage conditions. The counts found in the present investigation were lower, probably because evening milk was kept at the farm for approximately 4 h, versus 12 h.

The chemical composition of Parmigiano Reggiano is shown in Table 2 and Table 3, for the outer and inner zone of the wheel, respectively.

MC9 had lower fat and higher protein contents than MC20 (*p* ≤ 0.05), both in the outer and inner zones. Such differences, although statistically significant, were rather small and were not caused by the different moisture level, because they were confirmed by calculation on dry matter (*p* ≤ 0.05). The lower fat content was connected to the lower fat-to-casein ratio of the vat milk (1.01 vs. 1.05, respectively; *p* ≤ 0.05).

The fat and crude protein values found were in accordance with the literature data. Summer et al. [57] reported average contents of fat and crude protein of 28.4% and 33%, respectively; D’Incecco et al. [58] reported average values of 32.61 and 31.3 g/100 g in 24-month ripened cheese; Malacarne et al. [13] found, in 24-month ripened cheese, values of 30.47% and 32.41% in the outer zone and 29.94% and 31.52% in the inner zone. On the other hand, the moisture contents resulted in being lower than those reported by Malacarne et al. [31] in the outer (31.16%) and in inner zone (32.81%).

Results for cheese composition are also consistent with those reported by Mammi et al. [28], in a research study on the effect of monensin addiction in the cow’s diet on the characteristics of Parmigiano Reggiano cheese. Compared to our results for the outer zone of the wheel, these authors found higher values for moisture (30.85 vs. 30.75 g/100 g) and fat (33.79 vs. 32.95 g/100 g), whereas the protein content was 30.85 vs. 31.26 g/100 g. In contrast, the inner zone contained less moisture and more fat, whereas the protein content was almost the same.

### 3.2. Effect of the of Milk Storage Temperature on Cheese Proteolysis and Organic Acids Content

Table 4 and Table 5 show the proteolysis parameters and the organic acids contents in the outer and inner zones of the cheeses, respectively, whereas the counts of mesophilic lactic bacteria are shown in Table 6.

Total, soluble and peptone N, as well as the ratio of peptone N to total and to soluble N, showed significant differences between the two types of cheese, in both zones of the wheel (*p* ≤ 0.05). MC9 cheese had higher contents of total, soluble and peptone N, and also higher values of peptone N to total and to soluble N ratios than MC20 cheese. However, the ripening index was not statistically different, since MC9 cheese contained more total N. This finding is consistent with the similar content of total mesophilic lactic bacteria observed in the two theses.

Actually, the higher content of peptone N, as well as its ratio to both total and soluble N in MC9 cheese, suggests a faster primary proteolysis with respect to MC20. Despite its statistical significance, such a difference seems too small to affect typicality of the cheese, and it should be considered to be normal in an artisanal manufactured product as Parmigiano Reggiano is.

Results for proteolysis are roughly consistent with those reported by Mammi et al. [28] on Parmigiano Reggiano after 18 months of ripening. The values for total N (4.83–4.90 g/100 g) and soluble N (1.42–1.50 g/100 g) found in that investigation are very similar to those found in the present experimentation for the inner zone of the wheel, whereas our values in the outer zone are higher. Despite the difference in the ripening times, the ripening indices are rather similar (29.35 vs. 30.69).

The values of soluble and peptone N, and of peptone N to TN ratio and SN ratio observed in the cheese manufactured with milk stored at 9 °C agree with those reported by Pecorari et al. [59]. In a research study on 86 Parmigiano Reggiano cheese wheels aged 17–19 months, collected from 46 cheese factories, these authors reported average values of 1.56 mg/100 g and 0.20 mg/100 g for soluble N and peptone N, respectively; 4.03% for peptone N to TN ratio; and 13.21% for peptone N to SN ratio.

As for organic acids, the concentrations of all compounds were not statistically different between the two types of cheese. The content of pyruvic acid in the outer zone of both MC9 and MC20 cheeses was higher than that reported by Careri et al. [41] (5.1 mg/100 g). The same was for citric acid, whose concentration was higher than that reported by the same authors [41] (51 mg/100 g). This latter result suggests a slower degradation of citric acid in our experimentation, which is known to depend on the composition of microbiota, particularly on the presence of citrate-fermenting species or strains. It must be highlighted that citric acid was more abundant in the inner zone than in the outer one, suggesting a possible contribution to degradation by the surface microflora.

Results for acetic acid are consistent with data provided by Careri et al. [41] (99 mg/100 g) and, more recently, by Mammi et al. [28] (98.87 mg/100 g). On the other hand, propionic acid average values reported by Mammi et al. [28] (0.79–0.94 mg/100 g) are higher than our values, especially for the inner part of the wheel. It is worth mentioning that the propionic acid content should be as low as possible in Parmigiano Reggiano, since its presence is connected to texture defects and undesirable flavors [28].

## 4. Conclusions

In conclusion, storing milk at the farm, at 9 °C, significantly (*p* ≤ 0.05) influenced the chemical composition and the ripening process of Parmigiano Reggiano cheese. The most relevant influence regarded the fat and protein content: The former was lower, and the latter was higher, as compared to the cheese produced with milk stored at 20 °C, in compliance with the official manufacturing protocol. As far as proteolysis is concerned, only the peptone nitrogen content differed significantly, as it increased in the cheese produced with the milk stored at the lower temperature. Despite the statistical significance, the level of the differences recorded seems too small to affect typicality of the cheese, and might be considered as normal. In other terms, such a difference should be included within the ordinary range of the batch-to-batch and factory-to-factory variabilities.

## Figures and Tables

**Table 1:** Chemical composition and microbiological characteristics of the full cream evening milk kept at the herd at 9 °C (MC9) or 20 °C (MC20) (least-square-mean values).

Parameter	Unit	MC9 *n* ^1^ = 6	MC20 *n* ^1^ = 6	SE ^2^	*p* ^3^
Lactose	g/100 g	4.85	4.85	0.01	NS
Fat	g/100 g	3.55	3.51	0.01	NS
Protein	g/100 g	3.10	3.09	0.01	NS
Casein	g/100 g	2.41	2.41	0.01	NS
pH-values	Units	6.70	6.69	0.01	NS
Titratable acidity	SH/50 mL	3.24	3.28	0.01	NS
Somatic cell count	Log_10_(cells/mL)	5.41	5.43	0.03	NS
Total bacterial count	Log_10_(CFU/mL)	4.44	4.38	0.07	NS
Mesophilic lactic bacteria	Log_10_(CFU/mL)	3.15	3.20	0.06	NS
Psychrotrophs bacteria	Log_10_(CFU/mL)	2.70	3.00	0.09	*
Proteolytic bacteria	Log_10_(CFU/mL)	2.50	2.22	0.12	NS
Lipolytic bacteria	Log_10_(CFU/mL)	3.01	3.23	0.07	NS
Clostridia spores	Log_10_ (Spores/L)	1.51	1.25	0.09	NS

^1^ Number of samples. ^2^ Standard error. ^3^ *p*-value: NS, *p* > 0.05; * *p* ≤ 0.05.

**Table 2:** Chemical composition of the outer zone of cheese wheels produced by milk kept at the farm at 9 °C (MC9) or 20 °C (MC20) (least-square-mean values).

Parameter	Unit	MC9 *n* ^1^ = 6	MC20 *n* ^1^ = 6	SE ^2^	*p* ^3^
pH	Value	5.47	5.44	0.01	NS
Moisture	g/100 g	30.29	30.04	0.30	NS
Fat	g/100 g	29.67	30.40	0.33	*
Crude protein	g/100 g	32.94	31.93	0.37	*
Ash	g/100 g	4.34	4.19	0.06	NS
Salt (NaCl)	g/100 g	1.33	1.34	0.05	NS
Phosphorus	mg/100 g	660.86	637.13	7.86	NS
Calcium	mg/100 g	952.88	968.79	20.85	NS
Magnesium	mg/100 g	40.15	39.46	1.46	NS
Potassium	mg/100 g	273.19	224.09	9.50	NS
Fat	g/100 g of dry matter	42.57	43.45	0.34	*
Crude protein	g/100 g of dry matter	46.86	45.64	0.45	*
Salt (NaCl)	g/100 g of dry matter	1.91	1.92	0.07	NS

^1^ Number of samples. ^2^ Standard error. ^3^ *p*-value: NS, *p* > 0.05; * *p* ≤ 0.05.

**Table 3:** Chemical composition of the inner zone of cheese wheels produced by milk kept at the farm at 9 °C (MC9) or 20 °C (MC20) (least-square-mean values).

Parameter	Unit	MC9 *n* ^1^ = 6	MC20 *n* ^1^ = 6	SE ^2^	*p* ^3^
pH	Value	5.47	5.50	0.01	NS
Moisture	g/100 g	33.58	33.52	0.14	NS
Fat	g/100 g	27.97	28.62	0.26	*
Crude protein	g/100 g	31.59	30.81	0.23	*
Ash	g/100 g	3.97	3.92	0.07	NS
Salt (NaCl)	g/100 g	1.28	1.25	0.05	NS
Phosphorus	mg/100 g	611.68	605.60	6.05	NS
Calcium	mg/100 g	882.69	892.58	18.51	NS
Magnesium	mg/100 g	36.06	36.32	1.10	NS
Potassium	mg/100 g	190.27	172.11	7.54	NS
Fat	g/100 g of dry matter	42.12	43.02	0.35	*
Crude protein	g/100 g of dry matter	47.16	46.22	0.36	*
Salt (NaCl)	g/100 g of dry matter	1.93	1.88	0.07	NS

^1^ Number of samples. ^2^ Standard error. ^3^ *p*-value: NS, *p* > 0.05; * *p* ≤ 0.05.

**Table 4:** Proteolysis and organic acid contents in the outer zone of cheese wheels produced by milk kept at the farm at 9 °C (MC9) or 20 °C (MC20) (least-square-mean values).

Parameter	Unit	MC9 *n* ^1^ = 6	MC20 *n* ^1^ = 6	SE ^2^	*p* ^3^
Total N (TN)	g/100 g	5.16	5.01	0.06	*
Soluble N at pH 4.4 (SN)	g/100 g	1.59	1.49	0.02	*
Ripening index (SN/TN)	%	30.71	29.76	0.35	NS
Peptone N	g/100 g	0.19	0.15	0.02	*
Peptide N	g/100 g	0.28	0.26	0.03	NS
Amino acid N	g/100 g	1.00	0.97	0.02	NS
Ammonia N	g/100 g	0.11	0.11	0.01	NS
Peptone N/TN	%	3.59	2.94	0.38	*
Peptide N/TN	%	5.54	5.30	0.55	NS
Amino acid N/TN	%	19.37	19.32	0.45	NS
Ammonia N/TN	%	2.21	2.12	0.08	NS
Peptone N/SN	%	11.73	9.91	1.29	*
Peptide N/SN	%	18.00	17.89	1.81	NS
Amino acid N/SN	%	63.06	65.04	1.07	NS
Ammonia N/SN	%	7.20	7.15	0.23	NS
Lactic acid	g/100 g	1.52	1.57	0.05	NS
L-lactic acid	g/100 g	0.76	0.80	0.04	NS
D-lactic acid	g/100 g	0.76	0.78	0.02	NS
Acetic acid	mg/100 g	103.62	100.44	4.30	NS
Propionic acid	mg/100 g	0.41	0.65	0.11	NS
Pyruvic acid	mg/100 g	16.37	11.01	3.38	NS
Citric acid	mg/100 g	83.16	90.35	20.68	NS
Malic acid	mg/100 g	62.06	48.88	11.80	NS
Fumaric acid	mg/100 g	0.93	0.75	0.10	NS
Pyro-glutamic acid	mg/100 g	450.25	435.12	19.99	NS

^1^ Number of samples. ^2^ Standard error. ^3^ *p*-value: NS, *p* > 0.05; * *p* ≤ 0.05.

**Table 5:** Proteolysis and organic acid composition in the inner zone of cheese wheels produced by milk kept at the farm at 9 °C (MC9) or 20 °C (MC20) (least-square-mean values).

Parameter	Unit	9 °C (TS) *n* ^1^ = 6	20 °C (TC) *n* ^1^ = 6	SE ^2^	*p* ^3^
Total N (TN)	g/100 g	4.95	4.83	0.04	*
Soluble N at pH 4.4 (SN)	g/100 g	1.54	1.47	0.03	*
Ripening index (SN/TN)	%	31.13	30.50	0.51	NS
Peptone N	g/100 g	0.16	0.12	0.01	*
Peptide N	g/100 g	0.31	0.33	0.02	NS
Amino acid N	g/100 g	0.96	0.91	0.03	NS
Ammonia N	g/100 g	0.11	0.11	0.01	NS
Peptone N/TN	%	3.19	2.56	0.24	*
Peptide N/TN	%	6.27	6.79	0.33	NS
Amino acid N/TN	%	19.38	18.94	0.52	NS
Ammonia N/TN	%	2.23	2.17	0.10	NS
Peptone N/SN	%	10.27	8.43	0.80	*
Peptide N/SN	%	20.24	22.29	1.15	NS
Amino acid N/SN	%	62.34	62.16	0.96	NS
Ammonia N/SN	%	7.15	7.13	0.26	NS
Lactic acid	g/100 g	1.47	1.47	0.02	NS
L-lactic acid	g/100 g	0.80	0.78	0.03	NS
D-lactic acid	g/100 g	0.68	0.69	0.03	NS
Acetic acid	mg/100 g	98.36	99.67	4.43	NS
Propionic acid	mg/100 g	0.19	0.21	0.02	NS
Pyruvic acid	mg/100 g	7.23	5.21	1.50	NS
Citric acid	mg/100 g	128.62	112.17	15.57	NS
Malic acid	mg/100 g	31.25	27.74	6.56	NS
Fumaric acid	mg/100 g	0.59	0.58	0.04	NS
Pyro-glutamic acid	mg/100 g	410.05	437.25	21.71	NS

^1^ Number of samples. ^2^ Standard error. ^3^ *p*-value: NS, *p* > 0.05; * *p* ≤ 0.05.

**Table 6:** Mesophilic lactic bacteria content in the outer and inner zones of cheese wheels produced by milk kept at the farm at 9 °C (MC9) or 20 °C (MC20) (least-square-mean values).

Parameter	Unit	MC9 *n* ^1^ = 6	MC20 *n* ^1^ = 6	SE ^2^	*p* ^3^
Outer zone	Log_10_(CFU/g)	4.36	4.47	0.13	NS
Inner zone ^4^	Log_10_(CFU/g)	<2	<2		

^1^ Number of samples. ^2^ Standard error. ^3^ *p*-value: NS, *p* > 0.05. ^4^ Every samples show a value less than 100 CFU/g.

## Data Availability

The data presented in this study are available on request from the corresponding author.
